# Prior fluid and electrolyte imbalance is associated with COVID-19 mortality

**DOI:** 10.1038/s43856-021-00051-x

**Published:** 2021-11-25

**Authors:** Satu Nahkuri, Tim Becker, Vitalia Schueller, Steffen Massberg, Anna Bauer-Mehren

**Affiliations:** 1 Data Science, Pharma Research and Development, Roche Innovation Center Zurich, Zurich, Switzerland; 2 Data Science, Pharma Research and Development, Roche Innovation Center Munich, Munich, Germany; 3 Medizinische Klinik und Poliklinik I, Klinikum der Universität München, Munich, Germany

**Keywords:** Prognostic markers, Viral infection

## Abstract

**Background:**

The COVID-19 pandemic represents a major public health threat. Risk of death from the infection is associated with age and pre-existing comorbidities such as diabetes, dementia, cancer, and impairment of immunological, hepatic or renal function. It remains incompletely understood why some patients survive the disease, while others do not. As such, we sought to identify novel prognostic factors for COVID-19 mortality.

**Methods:**

We performed an unbiased, observational retrospective analysis of real world data. Our multivariable and univariable analyses make use of U.S. electronic health records from 122,250 COVID-19 patients in the early stages of the pandemic.

**Results:**

Here we show that a priori diagnoses of fluid, pH and electrolyte imbalance during the year preceding the infection are associated with an increased risk of death independently of age and prior renal comorbidities.

**Conclusions:**

We propose that future interventional studies should investigate whether the risk of death can be alleviated by diligent and personalized management of the fluid and electrolyte balance of at-risk individuals during and before COVID-19.

## Abstract (web summary)

Nahkuri et al. evaluate potential prognostic factors for COVID-19 mortality in a large US database of electronic health records. They find that fluid, pH and electrolyte imbalances – diagnosed at least one month prior to COVID-19 diagnosis – are associated with mortality.

## Plain language summary

The clinical course of patients with COVID-19 is highly variable, with some patients barely affected and others dying. We wanted to better understand why this is the case and identify markers of COVID-19-associated mortality. To this end, we looked into the entire available medical history of more than 100,000 COVID-19 patients from the United States. We found that patients who had experienced a disturbance of electrolyte or fluid levels in the year before they contracted SARS-CoV-2 were more likely to die than patients without such a history. This observation suggests that careful monitoring and balancing of the hydration and electrolyte status during and even before a SARS-CoV-2 infection may be beneficial and possibly reduce the risk of death with COVID-19.

## Introduction

In 2020, the world ground to a halt owing to the COVID-19 pandemic that still continues on its course in large parts of the world. Achieving durable universal sterilizing immunity through population-penetrating and transmission-halting vaccinations still remains a remote prospect: as of now, only 39.9% and 1.8% of the entire world population and that in low income countries, respectively, have started their COVID-19 immunization regimes^1,2^. Moreover, vaccine hesitancy percentages of up to 40% are reported in some large countries such as the U.S., Italy, and Russia^3^. As it seems possible that numerous COVID-19-naive individuals are yet to contract the virus, we anticipate that improvements in fortifying the health of risk group individuals in anticipation of a potential infection, as well as improving the outcomes of patients succumbing to the severe form of COVID-19, may translate to large savings in loss of life.

The SARS-CoV-2 virus that causes COVID-19 enters human cells via the ACE2 receptor. The infection first occurs in upper airways and at later stages may proceed to the lung, gastrointestinal tract, kidney, heart, or brain^4^. ACE2 and its antagonistic homolog ACE are core enzymes of the renin–angiotensin–aldosterone system (RAAS), which regulates electrolyte homeostasis, blood pressure, and cardiovascular health^5^, as well as restores balance upon volume disturbance of extracellular fluid^6,7^. The antagonistic effects of ACE and ACE2 are largely achieved by increases or decreases of the amount of circulating Angiotensin II, respectively. Angiotensin II is a potent secretagogue of aldosterone, an adrenal cortex hormone that enhances renal reabsorption of sodium and water, excretion of potassium, and the maintenance of acid–base balance^8,9^.

The underlying mechanisms of infection and viral spread are not fully understood, and despite the advances in prevention and treatment of severe COVID-19, an unmet need remains to better understand the clinical course and the risk factors of severe disease and death. Here, we present an agnostic and data-driven analysis of real world data from U.S. electronic health records (EHR) of 122,250 COVID-19 patients to identify a priori factors associated with death during a COVID-19 infection. Our unbiased analyses reveal pre-existing aberrations of fluid, pH or electrolyte levels as risk factors for COVID-19 mortality. We suggest that balancing electrolyte homeostasis in COVID-19 patients offers opportunities for better care and/or prevention of the severe disease.

## Methods

### Study design and participants

We extracted 122,250 COVID-19 cases collected by Optum^®^ with a diagnosis date between 20 February and 1 July 2020 (Supplementary Data 1). The Optum^®^ de-identified COVID-19 EHR dataset contains patient-level medical and administrative records from hospitals, emergency departments, outpatient centers, and laboratories from across the United States. Mortality information is derived from combining data from the Social Security Death Master File, hospital reports on patient deaths, and third party obituary sources. Data de-identification is performed in compliance with the HIPAA Expert Method and managed according to Optum^®^ customer data use agreements. The COVID-19 EHR dataset sources clinical information from hospital networks that provide data meeting Optum’s internal data quality criteria. We confirmed COVID-19 diagnosis either by documented ICD-10 codes (Supplementary Information: Data Preparation) or via positive PCR test result (Supplementary Data 2). Survival time was computed as the number of days between the date of COVID-19 diagnosis and last documented clinical activity (vitals, labs, medication, encounter, collected until 13 July 2020) or documented death.

We analyzed variables that were observed at least a month before the infection. We selected such a conservative buffer time period, since some patients might have had an undiagnosed COVID-19 infection for days before an opportunity to get tested. We also wanted to exclude any physiological changes potentially incurred by the virus during the incubation period, which may be up to 14 days^10^. We used the median value captured between 1 and 12 months before the initial COVID-19 diagnosis for vitals and laboratory measurements, and the entire past medical history (mean length ~5.4 years, Supplementary Data 1) for variables with a long-term effect, such as diagnoses of chronic indications. Prior inoculations were handled analogously.

We investigated all disease entities that are a part of the Charlson comorbidity index^11^, the AHRQ^12^, or the former Elixhauser definition^13^. A detailed description of assignment of ICD codes to disease entities can be found in Supplementary Data 3.

After quality control (Supplementary Information: Quality control, transformation, and handling of missing data), 249 variables were available for primary univariable analysis. For multivariable analysis, patients with an exaggerated proportion of missing data were removed, leaving 55,757 patients for analysis. Variables available for less than 10,000 patients were mean-imputed, reducing the overall missing rate from 22.1% to a remaining missingness rate of 12.4% in total. The remaining missing values were imputed using the missForest R-package^14^.

### Association analysis and model development

We originally set out to identify prognostic biomarkers that could identify patients at risk of COVID-19 mortality already before the onset of the disease. As primary analysis, we performed time-to-event analysis using Cox regression^15^. Univariable analysis was conducted using age, sex, ethnicity, race, insurance status, and US region/division as covariate parameters for adjustment. We applied a Bonferroni-correction with the number of variables (*m* = 249) to account for multiple testing and required a significance level of *α* = 0.05/m = 2 × 10^−4^. The univariable associations were calculated for the entire patient cohort, as well as separately for the age groups <50, 50–70, 70–80, and >80 years (Supplementary Data 4).

In order to allow comparison of hazard ratios (HRs) between different variables, we report the 2-standard-deviations hazard ratio “HR^2SD^”. It is computed as HR^2SD^ = HR^{2 × SD}^, where SD is the standard deviation of the respective variable.

As secondary analysis, multivariable modeling was performed. We pursued two approaches in parallel. First, we performed a backward selection procedure on the Cox regression model of all eligible variables. We iteratively removed the variable with least impact on model performance until all remaining parameters were significant at *α*_1_ = 0.05/249 = 2 × 10^−4^ (Bonferroni-correction). By construction, the procedure controls the family-wise error rate at *α* = 0.05. In parallel, we derived a regularized Lasso model^16^. We fitted a L1 (Lasso) regularized Cox-Proportional Hazards Model using glmnet version 3.02^16^, with the concordance index (C-index)^17^ as the performance measure. The regularization parameter *λ* was optimized using ten-fold cross-validation. We selected *λ* such that we extracted the most regularized model with a C-index within one standard error of the best performing model.

More details on model assumption checking, calibrations and performance measures can be found in Supplementary Information: Supplementary Methods and Supplementary Figs. S1 and S2.

### Ethical framework under which this study was conducted

Use of the Optum EHR data for research purposes has been determined by the New England Institutional Review Board (IRB) to not constitute research involving human subjects. This study has also been exempted from further IRB oversight in Switzerland and Germany by Kantonale Ethikkommission Kanton Zürich and Ethik-Kommission der Bayerischen Landesärztekammer, respectively.

The data licensed by Optum^®^ to support the study consists of only data de-identified in compliance with 45 CFR 164.514(a)-(c). The data has identifying information removed and is not coded in such a way that the data could be linked back to the subjects from whom it was originally collected.

The resulting research with this data would utilize data that did not include Human Subjects, as there is no interaction or intervention with living individuals, and neither can the provider of the data nor the recipient link the data with identifiable individuals, as defined in HHS regulation 45 CFR 46.102(f).

Our research involving the data licensed by Optum^®^ and described above, does not require an IRB review, as analyses with the data would not meet the definition of “research involving human subjects”.

### Reporting summary

Further information on research design is available in the Nature Research Reporting Summary linked to this article

## Results

### Univariable analysis of predisposition

We set out to detect prognostic biomarkers identifying patients at risk of death already before the onset of COVID-19. The overall mortality in our data set is 5.5%, which is well in line with the case-fatality ratio estimate of 5.89% for the USA in early 2020^18^. We first pursued a univariable analysis of 249 clinical variables observed at least a month before the initial diagnosis. We selected this conservative buffer time to exclude the effect of physiological changes incurred by the infection before the diagnosis or during the incubation period of up to 14 days^10^. We used the median value captured between 1 and 12 months before the initial COVID-19 diagnosis for vitals and laboratory measurements, and the entire past medical history (mean length ~5.4 years) for variables with a long-term effect, such as diagnoses of chronic indications. The univariable associations were calculated for the entire data set, as well as for the age groups <50, 50–70, 70–80, and >80 years separately (Supplementary Data 4).

The univariable analysis revealed 127 variables significantly associated (*P* < 0.0002) with mortality (Supplementary Data 4). As expected, age was the strongest prognostic factor, with a per-year hazard-ratio (HR) of 1.08 [1.077;1.084], and a two-standard-deviation hazard-ratio HR^2SD^ of 17.8 [16.3;19.5]. The HR^2SD^ measure can reflect the risk increase between, for instance, patients of age 50 versus 90 (=2 SD difference in age). Complementary to previous reports on biomarkers measured during COVID-19, we observed lower levels of a priori measurements of albumin, hemoglobin, calcium, HDL cholesterol, lymphocyte proportion, and lymphocyte to leukocyte ratio more frequently in deceased patients. Moreover, non-survivors had a higher likelihood of a history of elevated blood urea nitrogen, creatinine, blood glucose, respiratory rate, red cell distribution width, neutrophil percentage, neutrophil-to-lymphocyte ratio, and total white blood cell count.

**Fig. 1:**
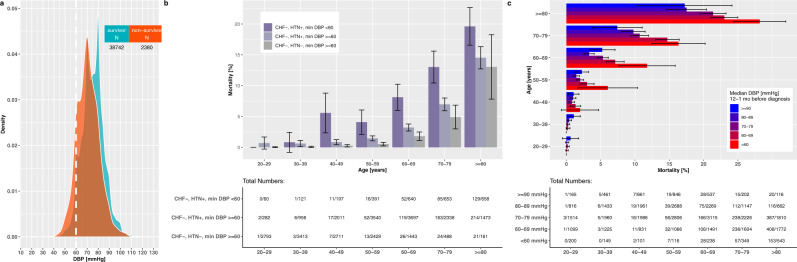
COVID-19 mortality is associated with prior measurements of abnormally low diastolic blood pressure (DBP). **a** Distribution of the median diastolic blood pressure measurement in COVID-19 survivors (cyan) versus non-survivors (orange) during days -365…-31 before the COVID-19 diagnosis date. Threshold for normal range, 60 mmHg, is shown as a white dotted line. **b** Mortality rates of patients without a recorded history of heart failure (HF-) and either with or without a history of hypertension (HTN + and HTN-, respectively). Cohorts had lowest diastolic blood pressure measurement either at <60 mmHg or > =60 mmHg on days -365…-31 before the infection. Patients are distinguished by age groups. 95% confidence intervals for the standard error are provided, and indicate the sampling error. **c** Mortality rates of patients as distinguished by age group and median diastolic blood pressure measurement levels on days -365…-31 before the infection. 95% confidence intervals for the standard error are provided, and indicate the sampling error. For the *N* = number of patients in each category, see the table inserts in the figure panels.

Unexpectedly, low rather than high measurements of diastolic blood pressure were associated with death (Supplementary Data 4, HR^2SD^ = 0.711 [0.655;0.771], *P* = 1.43E-16; Fig. 1a). This seemed to be in sharp contrast to earlier reports suggesting that hypertension was an important risk factor for COVID-19^19^. Curiously, a priori diagnoses of both hypotension (ICD-10 I95.1, I95.9) and hypertension (I.10*, O.100.*, O.109.*) were more common among the deceased patients (HR = 1.18 [1.14;1.22], *P* = 8.47E-24 and HR = 1.21 [1.14;1.29], *P* = 1.33E-09, respectively). Such ambivalent results might be associated with the natural decrease of DBP with age, or hypotension being a lagging comorbidity of heart failure, together with age and heart failure being risk factors for severe COVID-19^20^. However, after excluding all patients with a prior diagnosis of congestive heart failure and performing an age-group-specific analysis, we still observed an association of low DBP and mortality (Fig. 1b). Specifically, patients with a hypertension diagnosis (ICD-10 I.10*) and older than 40 years only showed consistently higher mortality rates, if they had also experienced abnormally low levels of DBP (<60 mmHg) in the past year. In fact, even abnormally high median measurements of DBP (>90 mmHg) were associated with lower mortality than abnormally low ones in the age groups 60–69 and 70–79 years (Fig. 1c). Indeed, the large epidemiological OpenSAFELY study^20^ has also previously reported that the fully adjusted variable of hypertension/elevated blood pressure has a negative rather than positive correlation with COVID-19 mortality, specifically in patients that are 70 years or older.

Another factor that could contribute to the hypotension observation is a decrease in serum albumin levels^21^. Decreased albumin levels were very highly associated with COVID-19 mortality in our data set, having the largest effect size for any laboratory measurement in the univariable analysis (HR^2SD^ = 0.471 [0.442;0.503], *P* = 5.10E-110). We also investigated the association of a priori measurements of DBP and median albumin levels, and observed that patients with abnormally low DBP typically had low albumin (OR^2SD^ = 0.23 [0.22, 0.24], *P* < 2e-16). We conclude that many Optum^®^ COVID-19 victims have a history of co-occurring low DBP and hypoalbuminemia.

### Multivariable analysis findings

To further dissect the predisposition landscape of COVID-19 mortality, we pursued a multivariable time-to-event analysis (Cox regression) of laboratory, vital, comorbidity, immunization, and demographic variables in 55,757 patients, for whom at least ten variables were available (see Supplementary Information). We created three multivariable models in parallel: one for comorbidities, one for laboratory measurements and vitals, and a combined main model for all variable groups as well as inoculations. Unless otherwise stated, we are discussing the combined multivariable model below.

The combined multivariable Cox model reaches a C-index of 0.853 (SE 0.003), a high level of prognostic power for survival (Supplementary Data 5). Both alternative models also showed good performances, with C-indices 0.841 (0.003) for the comorbidity model, and 0.851 (0.003) for the labs/vitals model. The combined model reveals a combination of factors that are well aligned with previous studies^20^ and associated with age, male sex, renal impairment, diabetes, hypoxia, hematological insult, dementia, and cancer (Table 1). As repeatedly reported, the strongest associations with mortality are observed for age (HR^2SD^ = 6.95 [6.11;7.91]) and male sex (HR^2SD^ = 1.82 [1.66; 1.98]). We also confirmed independent effects of African ethnicity and insurance status, as previously observed and discussed by Yehia et al.^22^. Furthermore, while an association of death during COVID-19 and increased red blood cell distribution width (RDW) had previously been reported^23^, our predisposition model shows that RDW aberrations precede the infection by at least a month.

**Table 1:** Combined multivariable model of a priori prognostic factors for COVID-19 mortality (*n* = 55,757), complemented with univariable results and potential clinical associations.

Variable	Multivariable model, *P*	HR 2 SD	LCL 2 SD	UCL 2 SD	Univariable model, *P*	HR 2 SD	LCL 2 SD	UCL 2 SD	*N*	Possible clinical associations
Age	2.54E-191	6.95	6.11	7.91	0.00E+00	17.8	16.3	19.5	122,250	complex
Male gender	7.31E-41	1.82	1.66	1.98	2.39E-100	1.76	1.67	1.86	122,250	complex
Albumin	1.56E-18	0.731	0.682	0.784	5.10E-112	0.471	0.442	0.503	46,191	renal
Red cell distribution width	7.29E-17	1.34	1.25	1.44	4.86E-82	1.84	1.73	1.96	50,295	hematological insult
Insurance status: uninsured	1.14E-16	1.26	1.19	1.33	2.69E-17	1.21	1.16	1.26	122,250	NA
Blood urea nitrogen	5.21E-14	1.33	1.23	1.43	2.58E-67	1.9	1.77	2.04	53,846	renal
Respiratory rate	6.43E-13	1.46	1.32	1.62	5.00E-43	2.21	1.97	2.47	51,169	hypoxia
Fluid, pH and electrolyte imbalance (FPEI)	9.67E-12	1.3	1.21	1.41	3.97E-76	1.51	1.45	1.58	122,250	homeostasis
African-American race	1.30E-11	1.29	1.2	1.38	3.27E-14	1.22	1.16	1.29	122,250	complex
Dementia	1.23E-08	1.15	1.09	1.2	8.84E-22	1.17	1.13	1.21	122,250	neurology
Hemoglobin A1C	1.44E-08	1.23	1.15	1.33	4.28E-15	1.48	1.35	1.64	27,169	diabetes
Metastatic carcinoma	1.49E-08	1.15	1.09	1.2	5.02E-11	1.11	1.08	1.15	122,250	cancer
Insurance status: medicare	2.96E-08	1.23	1.14	1.32	4.14E-10	1.16	1.11	1.22	122,250	NA
Oxygen saturation	1.41E-07	0.838	0.785	0.895	7.99E-10	0.818	0.767	0.872	64,154	hypoxia
Height	2.09E-07	0.79	0.723	0.864	6.89E-04	0.832	0.748	0.925	61,694	pulmonary disadvantage
Moderate or severe liver disease	1.46E-06	1.11	1.07	1.16	7.77E-27	1.17	1.14	1.21	122,250	hepatic
Triglycerides	8.04E-06	1.21	1.11	1.31	4.78E-09	1.38	1.24	1.53	31,566	diabetes
Carbon dioxide total	8.70E-06	0.867	0.814	0.923	5.43E-15	0.763	0.713	0.817	51,259	homeostasis
Congestive heart failure	1.08E-05	1.14	1.08	1.21	1.97E-58	1.34	1.29	1.38	122,250	cardiovascular
Boostrix DTP vaccine	1.77E-05	0.843	0.78	0.912	8.91E-15	0.784	0.737	0.833	122,250	cross-reactive immunity

The most striking novel finding of the multivariable model is a comorbidity group of diagnoses associated with fluid, pH and electrolyte imbalance (FPEI) (Table 1). Electrolyte and pH disturbances might obviously imply renal function impairment. However, renal comorbidities did not show an independent association in the combined multivariable model. An investigation of the co-dependence of different variables revealed that renal comorbidities were excluded from the model by correlated but stronger independent associations of age, albumin, and blood urea nitrogen measurements, as well as FPEI diagnoses and red blood cell distribution width (Supplementary Data 6). Moreover, our comorbidity-only model showed that the associations of FPEI and renal comorbidities were indeed independent (Supplementary Data 7). Interestingly, FPEI had the highest hazard ratio and the lowest *P* value in the comorbidity multivariable model (HR = 1.37 [1.27;1.49], and *P* = 1.71E-14). We conclude that while FPEI disorders in COVID-19 patients may often indicate suboptimal function of the kidneys, they do not always co-occur with a diagnosed renal comorbidity.

A priori levels of DBP did not show an independent association in the multivariable model. However, another dissection of variable co-dependence showed that it was not the history of congestive heart failure that sequestered DBP from the model. Instead, the combined associations of age, albumin, red cell distribution width, and blood urea nitrogen removed DBP from the model (Supplementary Data 6). Finally, the hypertension comorbidity group did not make it to any of the multivariable models, either. Instead, the most correlated variables that progressed to the model in its stead were age, albumin, FPEI, and Hemoglobin A1c (Supplementary Data 6).

### Fluid, pH and electrolyte imbalance (FPEI) co-morbidity group deep dive

**Fig. 2:**
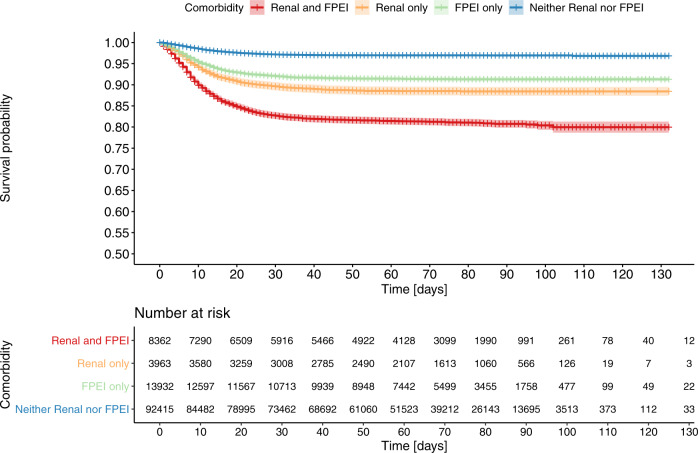
Overall survival of patients with a priori comorbidities associated with renal disorders and/or fluid, pH, and electrolyte imbalance (FPEI) as a Kaplan–Meier plot. Descriptors show overall survival of patients with either renal (orange) or FPEI (green) comorbidities, as well as patients with both (red) or neither (blue) comorbidities. For the *N* = number of patients at risk, see the table insert in the figure.

To further characterize the FPEI finding, we performed additional analyses involving both the prior medical history and the clinical findings during the COVID-19 infection. First, we inspected the overlap of patient cohorts with prior FPEI and/or renal comorbidities (Table 2). While the overlap was substantial, most patients with a history of FPEI did not have prior renal diagnoses. Interestingly, having a history of both renal and FPEI comorbidities was associated with a very high mortality, 25.0%. For comparison, mortalities of patient cohorts with renal only or FPEI only diagnoses were 15.2% and 10%, respectively. In fact, 26.0% of all non-survivors but only 6.0% of survivors in the entire data set had a combination of past FPEI and renal diagnoses. Moreover, an additional 19.5% of all deceased patients had an FPEI diagnosis but no renal diagnoses prior to the COVID-19 infection. From a Kaplan–Meier survival plot, a significant difference of survival can be observed for the four patient groups with/without renal/FPEI comorbidities (Fig. 2). Furthermore, we distinguished patients with end stage renal disease from other renal-diagnosed patients, but observed no significant difference (Supplementary Fig. S3).

**Table 2:** Mortality rates of patient cohorts with or without a prior history of fluid, pH and electrolyte imbalance (FPEI), and/or renal comorbidities.

	Neither FPEI nor renal history	Both FPEI and renal	Renal only	FPEI only	Total
Survivors	91903 (79.5%)	6967 (6.0%)	3603 (3.1%)	13059 (11.3%)	115532
Non-survivors	3117 (46.4%)	1745 (26.0%)	546 (8.1%)	1310 (19.5%)	6718
Mortality	3.4%	25.0%	15.2%	10.0%	5.8%

We repeated the univariable and multivariable analyses by including individual ICD codes from the FPEI comorbidity group (Supplementary Data 3). Two FPEI diagnoses made it to the comorbidity multivariable model: acidosis (ICD-10 E87.2), and mixed disorder of acid-base balance (E87.4) (Supplementary Data 7). Moreover, univariable analyses of all individual FPEI ICD codes showed a significant association with mortality (Supplementary Data 8). Lowest *P* values were observed for acidosis (E87.2), hyperkalemia (E87.5), and hypo-osmolality/hyponatremia (E87.1).

**Fig. 3:**
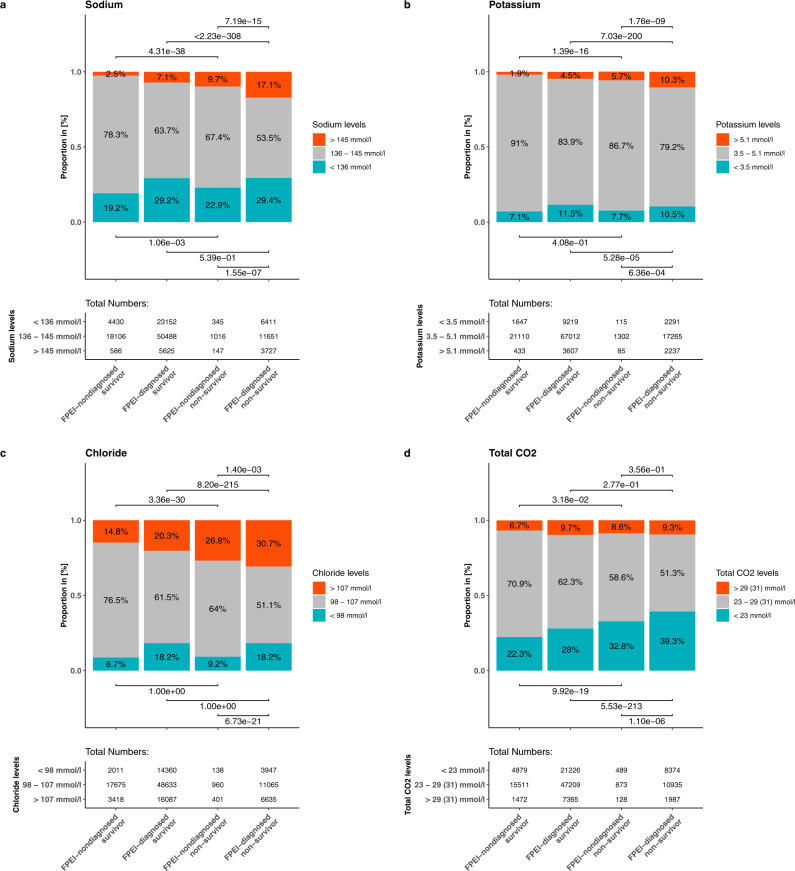
Measurements with abnormally high/low levels of electrolytes during a COVID-19 infection. Proportions of daily median measurements per patient, with normal (gray), abnormally high (orange), or abnormally low (cyan) levels of **a** sodium, **b** potassium, **c** chloride, or **d** total CO_2_ in the thirty days following the COVID-19 diagnosis date are shown. The four bars in each plot show measurements from survivors/non-survivors with/without a priori diagnoses with fluid, pH, and electrolyte imbalance (FPEI). Fisher’s Exact test used for pairwise comparison of categories, Bonferroni correction applied. For the *N* = number of median daily measurements in each category, see the table inserts in the figure panels.

We also investigated the laboratory values to detect disturbances in the levels of electrolytes or total CO_2_ in the thirty days following the COVID-19 diagnosis. We distinguished incidences of abnormally low or high potassium, sodium, chloride, or total CO_2_ in the patients’ median daily measurements by using thresholds for normal ranges from Healthline^24^. Among the non-FPEI-diagnosed cohort, non-survivors were more likely than survivors to have high potassium, sodium or chloride, or low sodium or total CO2 (Fig. 3a–d, pairwise comparisons between first and third bars). Within the FPEI-diagnosed cohort, non-survivors were more likely to have sodium, potassium or chloride levels above, or total CO2 levels below reference values (Fig. 3a–d, second versus fourth bars). Furthermore, all aberrations except high total CO_2_ were more frequent in non-survivors with a prior FPEI diagnosis than in non-survivors without one (Fig. 3a–d, third versus fourth bar). To sum it up, our analysis shows that both increased and decreased levels of sodium, chloride and potassium, and decreased levels of total CO_2_ are more frequent in COVID-19 non-survivors than in survivors.

**Fig. 4:**
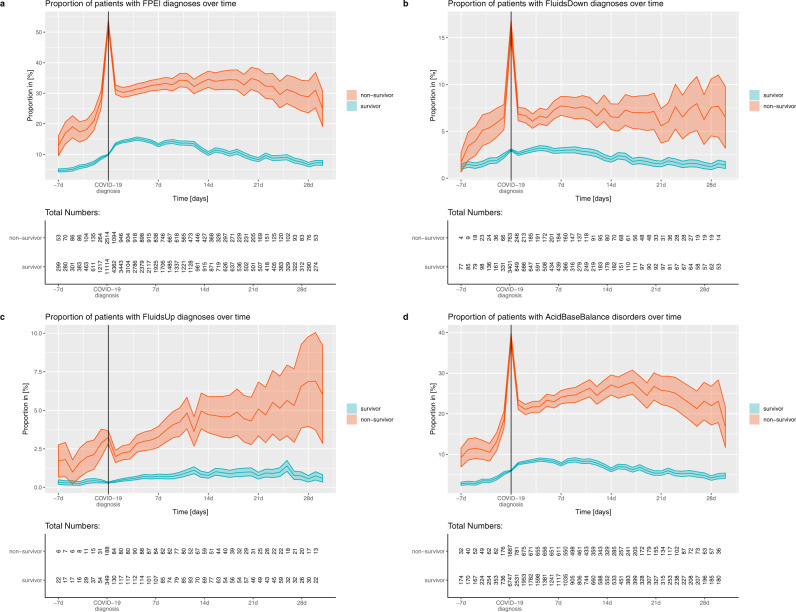
Cumulative incidence of fluid, pH, and electrolyte imbalance (FPEI) diagnoses assigned before and during COVID-19, separated by survivor versus non-survivor status. Proportion of patients having diagnoses associated with **a** any FPEI **b** fluid/volume deficit, ICD-10 codes E86.0, E86.1 or E86.9 **c** fluid/volume overload, ICD-10 codes E87.70 or E87.79 or **d** acid/base imbalance, ICD-10 codes E87.2, E87.3, E87.4, E87.5 or E87.6. The plot is split by survivor (cyan) versus non-survivor (orange) status. The underlying variable is a one-hot-encoded binary variable that indicates presence or absence of the comorbidity at the time point, “1” indicating presence, “0” indicating absence. The line shows the mean of the variable, i.e., the cumulative incidence. The standard error is shown as a shaded area around the mean, and indicates the sampling error. Plotted over days -7…+30 with respect to the COVID-19 diagnosis date. Non-survivors with an estimated death date before day +7 were excluded. For the *N* = number of patients with data available on each day, see the inserts in the figure panels.

Finally, we inspected the frequency of FPEI diagnoses assigned to the patients during COVID-19, and observed that FPEI diagnoses were significantly more common among the non-survivors (Fig. 4a). We also distinguished FPEI diagnoses to subgroups associated with volume/fluid depletion, fluid overload, or aberrations of pH, and observed each of these subcategories more frequently in non-survivors (Fig. 4b–d).

To summarize, we propose that a history of FPEI is a risk factor for COVID-19 mortality both in the absence of and coupled with pre-existing renal comorbidities.

## Discussion

Despite recent advances in COVID-19 vaccines and treatments, there is still an urgent need to better understand the course of disease and the risk factors of severe COVID-19. We have analyzed a rich real world data set of 122,250 COVID-19 patients and characterized the pre-existing clinical factors associated with risk of death. We foresee that our findings could generate novel hypotheses for COVID-19 treatment options and preventive medicine. The prognostic factors identified by our multivariable analysis are highly concordant with those from existing literature such as the large epidemiological OpenSAFELY study. This demonstrates that analyzing real world data to dissect COVID-19 predisposition is a powerful approach, and confirms that risk factors in European and American patients have a considerable overlap.

The multivariable and univariable models suggest multiple surprising associations. First, a previous inoculation with Diphtheria–Tetanus–Pertussis booster (NDC code 58160084252) is independently associated with lower risk of mortality in the combined multivariable model. Moreover, a group of herpes zoster vaccinations (NDC codes 00006496341, 58160082311, 58160081912, 00006496300) shows evidence of a protective effect in the univariable analysis, whereas a past diagnosis of herpes zoster is associated with lower mortality in the comorbidity multivariable model (Supplementary Data 4 and 7). Generally, it can be hypothesized that patients with active inoculation schedules have a keen interest in maintaining their health, and the financial means to pursue this goal. However, compelling computational evidence has been presented that the DTP vaccine harbors a number of T-cell epitopes with potential to induce cross-reactive immunity towards SARS-CoV-2^25^. Curiously, herpes zoster has been reported as a leading comorbidity of COVID-19^26^, as well as a recurring adverse effect of COVID-19 vaccination in rheumatic patients^27^. Second, the multivariable model suggests an independent association of low height with death (Table 1), in line with previous prospective observational studies indicating that height has an inverse correlation with mortality from respiratory and cardiovascular diseases^28,29^. Further studies will be necessary to identify clinical implications of these associations.

The most prominent novel outcome of our multivariable and univariable analyses is that a priori fluid, pH, and electrolyte imbalance (FPEI) is an independent predisposing factor for COVID-19 mortality. This may not be surprising considering that even mild electrolyte imbalance is associated with ill health and overall mortality^30^, but the predisposition association in the context of COVID-19 has not been previously reported to our best knowledge. Out of various individual FPEI comorbidities, the association of death with metabolic acidosis seems strongest, which is interesting considering previous observations of COVID-19 patients frequently developing acidosis or diabetic ketoacidosis^31^. All in all, both increases and decreases of sodium, potassium, chloride, and total CO_2_ demonstrate an association with death in our univariable analysis of lab values and co-morbidities from 1-12 months before the COVID-19 diagnosis, as well as measurements of electrolyte levels during the infection.

Fluid and electrolyte imbalance has previously been reported as a putative driver of adverse effects in critically ill patients submitted to ICU, and three underpinning mechanisms have been suggested: (1) reduced perfusion to the kidney owing to hypotension or hypovolemia, (2) tubular damage caused by ischemic or nephrotoxic kidney damage, and (3) inappropriate activation of kidney-regulating hormones such as those in the renin–angiotensin–aldosterone system^32^. It is well-fitting that our analyses recognize an association of COVID-19 mortality with FPEI, hypotension, albumin deficiency, and renal comorbidities alike. It is hence worthwhile in our opinion to ask whether hormonal factors of electrolyte homeostasis could also be involved.

Notably, ACE2, which is the entry point of SARS-CoV-2 into human cells, is also a core factor of the renin–angiotensin–aldosterone system (RAAS) that regulates electrolyte homeostasis. A large proportion of active ACE2 normally resides in the lung alveoli, while abundant presence is also observed in the heart, gastrointestinal tract, nasopharyngeal region, and vascular endothelium, and limited expression in many other tissues including brain, liver, and kidney^33,34^. Such widespread expression of ACE2 may also partly explain the variety of COVID-19 clinical symptoms. The effect of ACE2 on homeostasis is largely achieved by reducing the amount of circulating aldosterone, an adrenal cortex hormone that stimulates kidney principal cells and alpha intercalated cells to enhance renal reabsorption of sodium and water, excretion of potassium, and the maintenance of acid–base balance^8,9^. ACE2 is counterbalanced by the homolog ACE, as well as the antidiuretic hormone and the natriuretic peptide, which may also become activated upon electrolyte or pH imbalance^35,36^. Disturbance of ACE2 may contribute to COVID-19 clinical characteristics via its effects on regulating vasodilation and dampening inflammation^37^. However, the full consequences of ACE2 aberrations for electrolyte homeostasis during COVID-19 remain to be understood.

The role of all-round electrolyte and pH imbalance in COVID-19 mortality was partially unappreciated in some earlier studies that focused on the median laboratory measurements and hence averaged out the effects of opposite extremities^38,39^. Nevertheless, recent reports have pointed out that electrolyte imbalance in general^40,41^ and dysnatremia in particular are frequently observed during COVID-19^42–45^. Our prognostic models suggest that the presence of electrolyte imbalance more than a month before the onset of the infection may sensitize the patient to unfavorable outcomes from COVID-19.

Another striking finding of our predisposition models is that of hypoalbuminemia, which is also often associated with abnormally low levels of blood pressure and highly correlated with low diastolic blood pressure in our data set. Previous studies have indicated that a large majority of patients that are critically ill with COVID-19, and almost all non-survivors, either present with hypoalbuminemia at the start of the infection or develop it during the course of the disease^46^. Low serum albumin is also associated with overall mortality in the elderly^47^. Patients with low albumin will lose colloidal osmotic (oncotic) pressure, and experience an extracellular fluid shift from the intravascular space into the extravascular space^48^. Notably, hypoalbuminemia coupled with excess extracellular fluid may result in co-occurring edema and hypovolemia^49^. Such a mechanism has also been suggested to contribute to COVID-19 severity^50^, and would be particularly disastrous in the lung. Indeed, compelling suggestions have been presented that volume contraction may be aggravating COVID-19 outcomes^51^.

Our analysis is based on a real world data resource and thus carries the burden of associated difficulties and limitations. In contrast to controlled randomized settings, systematic bias and negative effects by data errors cannot be completely ruled out a priori. On the other hand, the large sample size available can help to overcome issues and to detect phenomena, which are otherwise overlooked.

Specifically, while inpatient deaths are captured with high certainty, death events in care homes facilities may be underreported to an unknown degree. Nevertheless, time from diagnosis to death events shows the typical skewed distribution in our data, with a relatively elevated portion of patients who die more than two months after the infection. Moreover, the mortality percentage we observe in our data set is similar to that reported in earlier literature. In general, it is highly likely that any possible underreporting of death cases will not invalidate our multivariable model, but rather results in underestimation of effect sizes. In other words, some effects are likely to be higher than reported here, but, given the high sample size, were still detectable.

Another limitation is that of blood pressure measurements. While they are typically expected to be captured via a manual method by the medical assistant at the point of care, the possibility cannot be excluded that at some facilities and some situations, they might be recorded by automated oscillometric systems. Should this be the case, the algorithmically calculated diastolic blood pressure values might introduce artefacts in the DBP variable.

Finally, the majority of risk factors we identify are consistent with previous findings in the literature, while results for comorbidities from patient history are consistent with those of biomarkers of the respective diseases, demonstrating internal consistency and plausibility of our findings. In summary, while the exact risk effect size might be influenced by the way data was collected, it is likely that the qualitative conclusions we provide are correct.

ICU deaths are often associated with challenges in maintaining electrolyte homeostasis^32^. Our findings complement this picture for COVID-19 patients by showing that the balance of fluid, pH and electrolytes is more frequently disturbed in victims than survivors at least a month before the onset of the infection, and associated with an increased risk of death independently of age and prior renal comorbidities. Future observational and interventional studies may show that careful and personalized correction of fluid and electrolyte homeostasis early in the course of the infection, or even before it, correlates with better outcomes of COVID-19.

## Supplementary information

Supplementary Information

Supplementary Data 1

Supplementary Data 2

Supplementary Data 3

Supplementary Data 4

Supplementary Data 5

Supplementary Data 6

Supplementary Data 7

Supplementary Data 8

Description of Additional Supplementary Files

## Data Availability

Source data for the main findings of the manuscript can be accessed as Supplementary Data 1–8. All data that support the findings of this study are available from Optum^*®*^, which owns the data. However, restrictions apply to the availability of these data, which were used under license for the current study, and so are not publicly available. Readers can pursue access to the data by contacting www.optum.com/lifesciences.

## Code availability

All statistical analyses were implemented in R as described in Methods and Supplementary Methods in Supplementary Information. The source code is deposited in 10.5281/ZENODO.5336101^52^.

## References

[1] Mathieu, E. et al. A global database of COVID-19 vaccinations. *Nat. Hum. Behav*. 10.1038/s41562-021-01122-8 (2021). 10.1038/s41562-021-01122-8 33972767

[2] Our World in Data, Coronavirus Vaccinations website (https://ourworldindata.org/covid-vaccinations, accessed 3 September 2021). (2021).

[3] Sallam M (2021). COVID-19 vaccine hesitancy worldwide: a concise systematic review of vaccine acceptance rates. Vaccines.

[4] Gupta A (2020). Extrapulmonary manifestations of COVID-19. Nat. Med..

[5] Patel VB, Zhong J-C, Grant MB, Oudit GY (2016). Role of the ACE2/angiotensin 1–7 axis of the renin–angiotensin system in heart failure. Circ. Res..

[6] Fountain, J. H. & Lappin, S. L. *Physiology, Renin Angiotensin System* (StatPearls Publishing, 2020).

[7] Navar LG (2014). Physiology: hemodynamics, endothelial function, renin–angiotensin–aldosterone system, sympathetic nervous system. J. Am. Soc. Hypertens..

[8] Atlas SA (2007). The renin-angiotensin aldosterone system: pathophysiological role and pharmacologic inhibition. J. Manag. Care Pharm..

[9] Wagner CA (2014). Effect of mineralocorticoids on acid-base balance. Nephron Physiol..

[10] Lauer SA (2020). The incubation period of Coronavirus Disease 2019 (COVID-19) from publicly reported confirmed cases: estimation and application. Ann. Intern. Med..

[11] Quan H (2011). Updating and validating the charlson comorbidity index and score for risk adjustment in hospital discharge abstracts using data from 6 countries. Am. J. Epidemiol..

[12] Agency for Healthcare Research and Quality. Elixhauser Comorbidity Software for ICD-10-CM Healthcare Cost and Utilization Project. (2018).

[13] Elixhauser A, Steiner C, Harris D, Coffey R (1998). Comorbidity measures for use with administrative data. Med. Care.

[14] Stekhoven DJ, Buhlmann P (2012). MissForest–non-parametric missing value imputation for mixed-type data. Bioinformatics.

[15] Enderlein G, Cox DR, Oakes D (1987). Analysis of Survival Data. Chapman and Hall, London – New York 1984, 201 S., £ 12,–. Biom. J..

[16] Simon N, Friedman J, Hastie T, Tibshirani R (2011). Regularization paths for Cox’s proportional hazards model via coordinate descent. J. Stat. Softw..

[17] Steck, H., Krishnapuram, B. & Dehing-Oberije, C. On Ranking in Survival Analysis: Bounds on the Concordance Index. (2008).

[18] Hoffmann, C. & Wolf, E. Older age groups and country-specific case fatality rates of COVID-19 in Europe, USA and Canada. *Infection.*10.1007/s15010-020-01538-w (2020). 10.1007/s15010-020-01538-w PMC7585357 33098532

[19] Zuin M (2020). Arterial hypertension and risk of death in patients with COVID-19 infection: systematic review and meta-analysis. J. Infect..

[20] Williamson EJ (2020). Factors associated with COVID-19-related death using OpenSAFELY. Nature.

[21] Høstmark AT, Tomten SE, Berg JE (2005). Serum albumin and blood pressure: a population-based, cross-sectional study. J. Hypertens..

[22] Yehia BR (2020). Association of race with mortality among patients hospitalized with Coronavirus Disease 2019 (COVID-19) at 92 US hospitals. JAMA Netw. Open.

[23] Foy BH (2020). Association of red blood cell distribution width with mortality risk in hospitalized adults with SARS-CoV-2 infection. JAMA Netw. Open.

[24] Healthline Basic Metabolic Panel (https://www.healthline.com/health/basic-metabolic-panel#normal-results), accessed 1 June 2021. (2017).

[25] Reche PA (2020). Potential cross-reactive immunity to SARS-CoV-2 from common human pathogens and vaccines. Front. Immunol..

[26] Elsaie, M. L., Youssef, E. A. & Nada, H. A. Herpes zoster might be an indicator for latent COVID-19 infection. *Dermatol. Ther*. **33**, e13666 (2020). 10.1111/dth.13666 PMC7267085 32447801

[27] Furer, V. et al. Herpes zoster following BNT162b2 mRNA Covid-19 vaccination in patients with autoimmune inflammatory rheumatic diseases: a case series. *Rheumatology.*10.1093/rheumatology/keab345 (2021). 10.1093/rheumatology/keab345 PMC8083327 33848321

[28] Smith GD (2000). Height and risk of death among men and women: aetiological implications of associations with cardiorespiratory disease and cancer mortality. J. Epidemiol. Community Health.

[29] Barker DJP, Osmond C, Golding J (1990). Height and mortality in the counties of England and Wales. Ann. Hum. Biol..

[30] Liamis G (2013). Electrolyte disorders in community subjects: prevalence and risk factors. Am. J. Med..

[31] Li J (2020). COVID‐19 infection may cause ketosis and ketoacidosis. Diabetes Obes. Metab..

[32] Lee JW (2010). Fluid and electrolyte disturbances in critically ill patients. Electrolytes Blood Press.

[33] Hamming I (2004). Tissue distribution of ACE2 protein, the functional receptor for SARS coronavirus. A first step in understanding SARS pathogenesis. J. Pathol..

[34] Chen, J. et al. Individual variation of the SARS‐CoV‐2 receptor ACE2 gene expression and regulation. *Aging Cell* **19**, e13168 (2020). 10.1111/acel.13168 PMC7323071 32558150

[35] Reid, I. A., Schwartz, J., Ben, L., Maselli, J. & Keil, L. C. In *Progress in Brain Research* Vol. 60 (Elsevier, 1983). 10.1016/S0079-6123(08)64414-3 6364215

[36] Richards AM (1996). The renin-angiotensin-aldosterone system and the cardiac natriuretic peptides. Heart.

[37] Groß S, Jahn C, Cushman S, Bär C, Thum T (2020). SARS-CoV-2 receptor ACE2-dependent implications on the cardiovascular system: from basic science to clinical implications. J. Mol. Cell. Cardiol..

[38] Lippi G, South AM, Henry BM (2020). Electrolyte imbalances in patients with severe coronavirus disease 2019 (COVID-19). *Ann. Clin*. Biochem. Int. J. Lab. Med..

[39] Chen, D. et al. *Hypokalemia and Clinical Implications in Patients with Coronavirus Disease 2019 (COVID-19)*. Preprint at *medRxiv*10.1101/2020.02.27.20028530 (2020).

[40] De Carvalho, H. et al. Electrolyte imbalance in COVID-19 patients admitted to the emergency department: a case–control study. *Intern. Emerg. Med*. 10.1007/s11739-021-02632-z (2021). 10.1007/s11739-021-02632-z PMC7823192 33484453

[41] Malieckal, D. A., Uppal, N. N., Ng, J. H., Jhaveri, K. D. & Hirsch, J. S. Electrolyte abnormalities in patients hospitalized with COVID-19. *Clin. Kidney J*. 10.1093/ckj/sfab060 (2021). 10.1093/ckj/sfab060 PMC7989524 34079619

[42] Hu, W. et al. Disorders of sodium balance and its clinical implications in COVID-19 patients: a multicenter retrospective study. *Intern. Emerg. Med*. (2020) 10.1007/s11739-020-02515-9. 10.1007/s11739-020-02515-9 PMC7563904 33064253

[43] Hirsch, J. S. et al. Prevalence and outcomes of hyponatremia and hypernatremia in patients hospitalized with COVID-19. *Nephrol. Dial. Transplant*. 10.1093/ndt/gfab067 (2021). 10.1093/ndt/gfab067 PMC7989196 33724428

[44] Tzoulis P (2021). Dysnatremia is a predictor for morbidity and mortality in hospitalized patients with COVID-19. J. Clin. Endocrinol. Metab..

[45] Frontera JA (2020). Prevalence and impact of hyponatremia in patients with Coronavirus Disease 2019 in New York city. Crit. Care Med..

[46] Huang W (2020). Decreased serum albumin level indicates poor prognosis of COVID-19 patients: hepatic injury analysis from 2,623 hospitalized cases. Sci. China Life Sci..

[47] Corti M-C (1994). Serum albumin level and physical disability as predictors of mortality in older persons. JAMA J. Am. Med. Assoc..

[48] Gounden, V., Vashisht, R. & Jialal, I. *Hypoalbuminemia* (StatPearls Publishing, 2020). 30252336

[49] Bobkova I, Chebotareva N, Kozlovskaya L, Shilov E (2016). Edema in renal diseases – current view on pathogenesis. Nephrol. Point Care.

[50] Huang J (2020). Hypoalbuminemia predicts the outcome of COVID‐19 independent of age and co‐morbidity. J. Med. Virol..

[51] Tsolaki V, Zakynthinos GE, Mantzarlis K, Makris D (2020). Increased mortality among hypertensive COVID-19 patients: pay a closer look on diuretics in mechanically ventilated patients. Heart Lung.

[52] Supplementary Software. *Covid19Code*. 10.5281/ZENODO.5336101 (Zenodo, 2021).

