# Supplementary material for: Prior fluid and electrolyte imbalance is associated with COVID-19 mortality
Source: Commun Med (Lond). 2021 Nov 25;1:51. doi: 10.1038/s43856-021-00051-x (PMC9053234; doi:10.1038/s43856-021-00051-x)
Supplement: Supplementary file 1 — Supplementary Information [file 43856_2021_51_MOESM1_ESM.pdf]

Supplementary Information

Supplementary Methods

Data preparation

We extracted 122,250 COVID-19 cases with a diagnosis date between 1 Feb 2020 and 1 Jul 2020 (Supplementary Data 1). Diagnosis was confirmed either by documented ICD10 codes in the OPTUM® diagnosis table any one of U07.1, U07.2, U07.3 or a combination of one of J12.89/J20.8/J40/J22/J98.8/J80 plus B97.29 on the same encounter or, alternatively, via positive test result for PCR laboratory tests (Supplementary Data 2). Survival status was derived from the OPTUM® patient table. Survival time was computed as the number of days between the date of COVID-19 diagnosis and last documented clinical activity (vitals, labs, medication, encounter).

Our analysis focused on predisposition. We kept all laboratory and vital data from a time window between 12 months prior to COVID-19 diagnosis to 1 month prior to COVID-19 diagnosis. In case of multiple measurements taken in the time window, the median value per patient was used. Medical history (MH), as documented by ICD codes, was included for the entire patient history available 1 month or more before the COVID-19 diagnosis. We defined indicator variables of whether a patient had or had not a prior history of a particular disease entity. We investigated all disease entities that a part of the Charlson comorbidity index[1], the AHRQ[2], Arrhythmia from the former Elixhauser definition[3], as well as a series of entities previously implicated in COVID-19 outcomes, including COPD, hypotension, and asthma. A detailed description of assignment of ICD codes to disease entities can be found in Supplementary Data 3. Prior immunizations were handled analogously.

Quality control, transformation and handling of missing data

In the first step, we kept all lab and vital parameters, which were available for at least 1,000 patients, leaving 249 variables for primary analysis. We set implausible measurements to missing (e.g. negative values, percentages greater than 100, "0" if not biologically sensible, etc.). For continuous variables, observations more than +/-3 interquartile ranges from the median were set to missing, unless more extreme values were deemed to be plausible according to the judgement of an experienced physician. We performed log-transformation of variables when the Shapiro-Wilk test[4] showed less significant deviation from normality on the log-scale than it did on the original scale. We confirmed the decision on transformation via visual inspection of the density plots. The accordingly processed and quality-controlled data were used for univariate association analysis (cf. below).

For multivariate analysis, we additionally removed all patients from the analysis, for which less than 10 different variables were available, leaving 55,757 patients for analysis. Variables available for less than 10,000 patients were mean-imputed, resulting in a remaining missing rate

of 12.4% in total. Those remaining missing values were imputed using the missForest R-package[5]. The package iteratively imputes missing values, and is suited for mixed-type data. It averages over unpruned trees, using the built-in out-of-bag error estimates of random forest.

We computed pairwise Pearson correlation coefficients[6] of variables, for the purpose of sanity checking of variables via to be expected correlations, and to investigate potential collinearity.

Association analysis

As primary analysis, we performed survival analysis, in particular Cox regression[7], to analyze time-to-death from the date of COVID-19 diagnosis. Univariate analysis was conducted using age, sex, ethnicity, race, insurance status, and US region/division as covariate parameters for adjustment. We applied a Bonferroni-correction with the number of variables (m=250) to account for multiple testing and judged variable association to be significant if it met an α-level of $\alpha=0.05/m=2*10^{-4}$. We used the R survival package[8] and in particular the survfit() function for survival times and the coxph() function for Cox regression. Median survival times were computed using the methodology as described in[9] and visualized as Kaplan-Meier plots[10]. For our main model, we computed Martingale residual[11] and visualized them as suggested in[12]. An interpolation curve was fitted with the R lowess() function[13]. We tested if the Cox proportional hazards (PH) assumption[14] was fulfilled and computed Schoenfeld residuals[15].

In order to allow comparison of, by default scale-dependent, hazard ratios (HRs) between different variables, we report the 2-standard-deviations hazard ratio “HR_2SD”. It is computed as HR_2SD=HR^{2*SD}, where SD is the standard deviation of the respective variable. Note that for a binary variable, the usual HR and HR_2SD coincide if the two binary outcomes are about equally frequent (e.g. for sex) and that HR_2SD is numerically smaller than the standard HR when the binary outcomes differ in frequency. This effect makes the HR_2SD more comparable to HRs for quantitative variables and implies also a stronger accordance with the actual prognostic power of the variable: for binary variables where one outcome is very rare, the impact on prognosis is comparatively smaller than for a binary variable with two equally frequent outcomes, since only a small portion of patients has the rare variable status and has the elevated risk. Using HR_2SD instead of HR compensates for this effect.

The association analysis between a priori measurements of hypotensive DBP and a priori median albumin levels (n=45,819 patients) was performed using logistic regression. To be consistent with the Cox regression the results are shown as 2-standard-deviations odds ratio “OR_2SD”.

Model development

As secondary analysis, multivariable modelling was performed. We pursued two approaches in parallel. First, we performed a backward selection procedure on the Cox regression model of all

eligible variables. We iteratively removed the variable with least impact on model performance, until all remaining parameters were significant at $\alpha_1=0.05/250=2*10^{-4}$ (Bonferroni-correction, two-sided). By construction, the procedure controls the family-wise error rate at $\alpha=0.05$. In parallel, we derived a regularized Lasso model[16]. We fitted a L1 (Lasso) regularized Cox-Proportional Hazards Model using glmnet version 3.02[16], with the concordance index (C-index)[17] as the performance measure. The regularization parameter λ was optimized using ten-fold cross-validation. We selected λ such that we extracted the most regularized model with a C-index within one standard error of the best performing model.

To compare model performance between the two approaches, we used the C-index and and receiver operating characteristics area under the curve (ROC-AUC) for censored data, implemented in the R package timeROC[18]. ROC-AUC was computed for different time points (5, 10, 20, 30, 60s-days survival).

For the final model, we checked if recently suggested requirements for developing multivariable prediction models were fulfilled[19].

Supplementary Results

The plot of the martingale residuals of the multivariate model (Supplementary Figure S1) and the red fitting line, which increases very slightly and only at the end, suggest that linearity on the log hazard ratio scale is a reasonable model assumption. Also the Schoenfeld residuals (Supplementary Figure S2) are distributed symmetrically, and do not suggest relevant systematic deviations from the proportional hazards assumption.

Supplementary Figure S1: Martingale residuals of the combined multivariate model with lowess fitting line

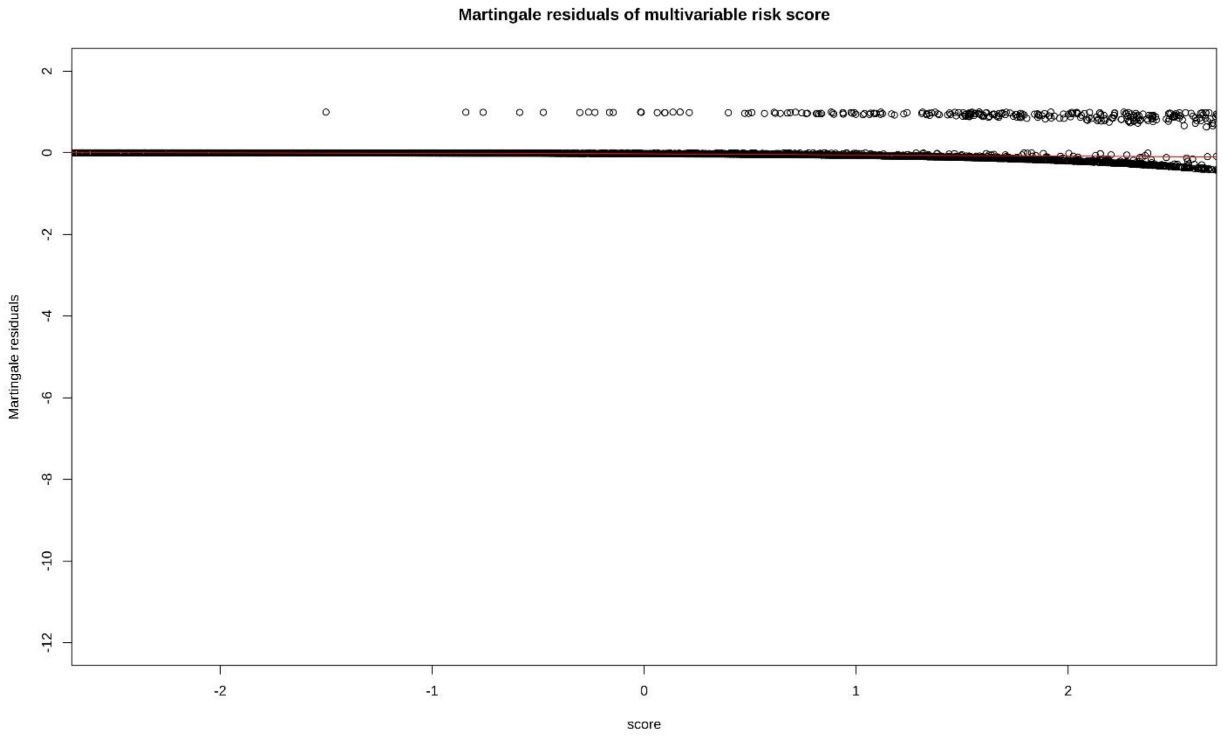

Supplementary Figure S2: Schoenfeld residuals of the combined multivariate model

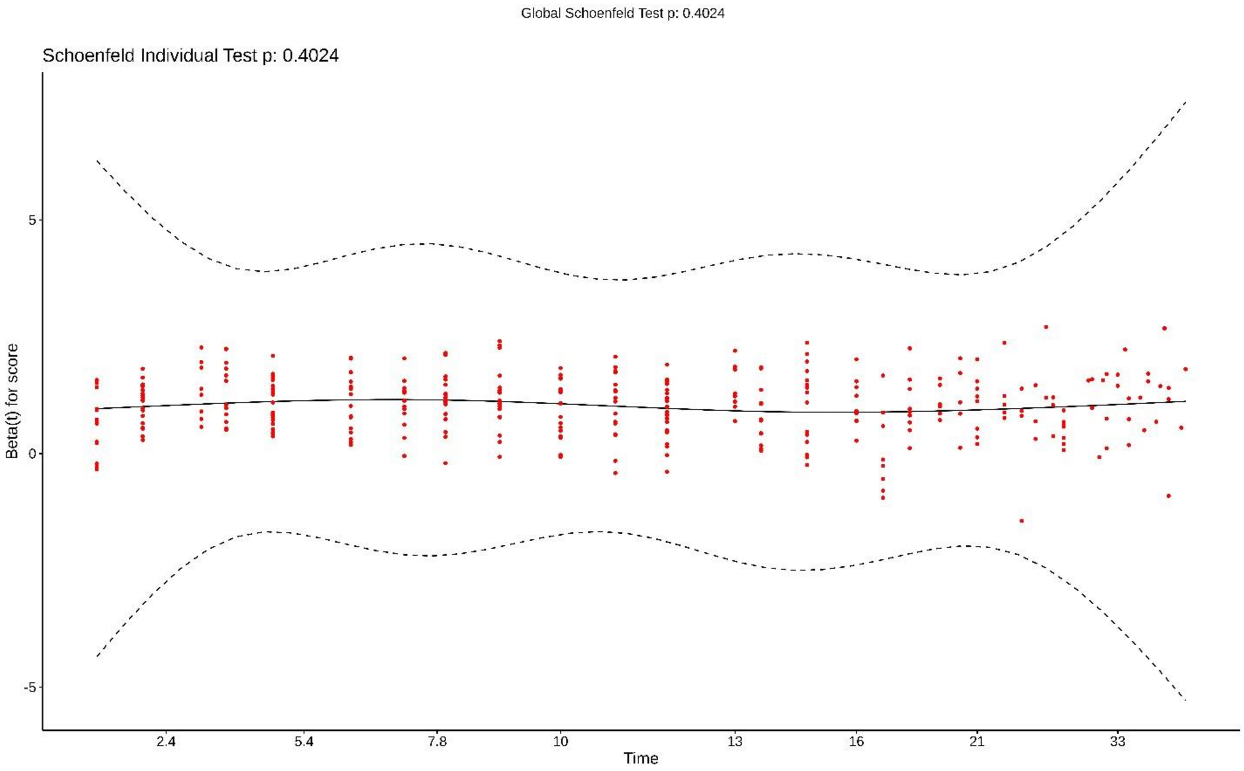

Supplementary Figure S3: Mortality of patients with *a priori* comorbidities associated with end stage renal disease (ESRD), other renal comorbidities, and/or fluid, pH and electrolyte imbalance (FPEI).

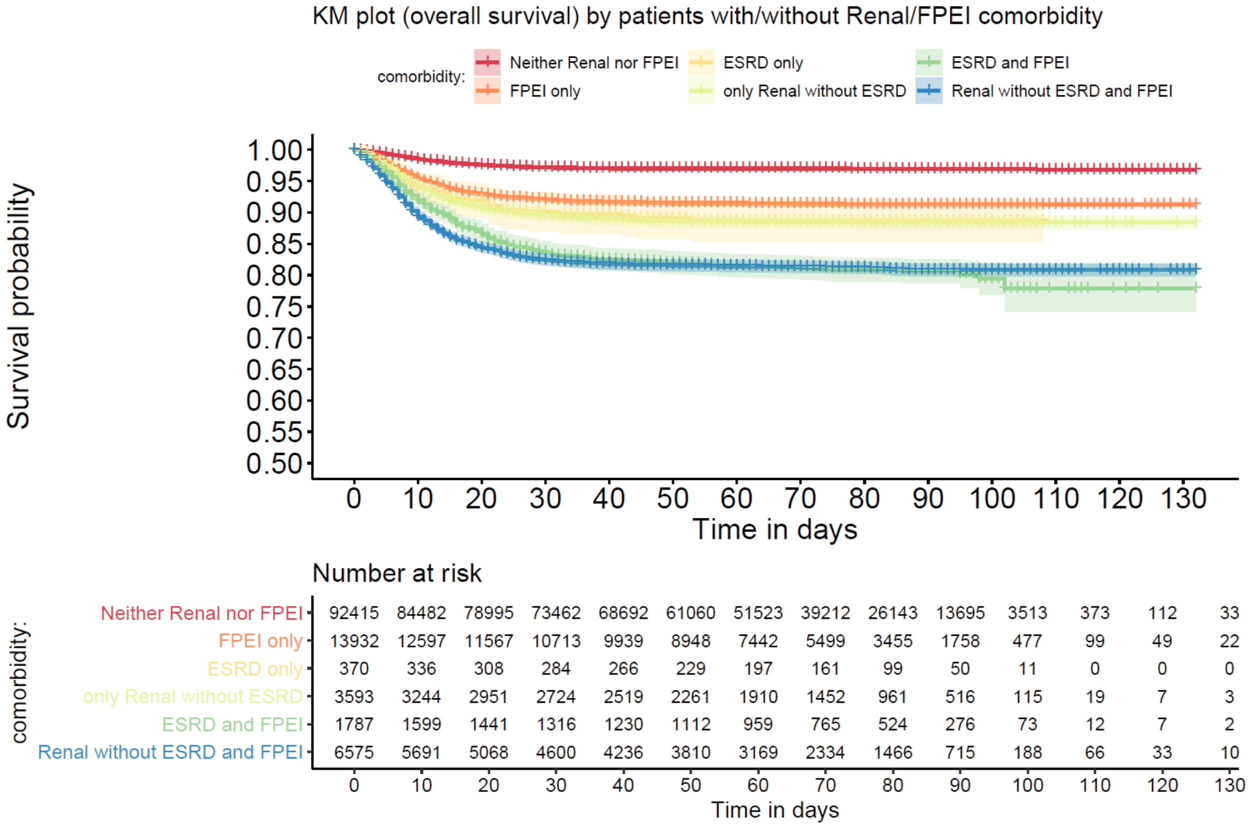

Supplementary References

1. Quan H, Li B, Couris CM, et al. Updating and Validating the Charlson Comorbidity Index and Score for Risk Adjustment in Hospital Discharge Abstracts Using Data From 6 Countries. *Am J Epidemiol*. 2011;173(6):676-682. doi:10.1093/aje/kwq433
2. Agency for Healthcare Research and Quality. Elixhauser Comorbidity Software for ICD-10-CM Healthcare Cost and Utilization Project. Published online 2018. Accessed April 23, 2018. https://www.hcup-us.ahrq.gov/toolssoftware/comorbidityicd10/comorbidity_icd10.jsp
3. Elixhauser A, Steiner C, Harris D, Coffey R. Comorbidity Measures for Use with Administrative Data. *Med Care*. 1998;36(1):8-27.
4. Shapiro SS, Wilk MB. An Analysis of Variance Test for Normality (Complete Samples). *Biometrika*. 1965;52(3/4):591. doi:10.2307/2333709
5. Stekhoven DJ, Buhlmann P. MissForest--non-parametric missing value imputation for mixed-type data. *Bioinformatics*. 2012;28(1):112-118. doi:10.1093/bioinformatics/btr597
6. VII. Note on regression and inheritance in the case of two parents. *Proc R Soc Lond*. 1895;58(347-352):240-242. doi:10.1098/rspl.1895.0041
7. Enderlein G. Cox, D. R.; Oakes, D.: Analysis of Survival Data. Chapman and Hall, London – New York 1984, 201 S., £ 12,–. *Biom J*. 1987;29(1):114-114. doi:10.1002/bimj.4710290119

8. Therneau T, Grambsch P. *Modeling Survival Data: Extending the Cox Model*. Springer-Verlag; 2000.
9. Kalbfleisch JD, Prentice RL. *The Statistical Analysis of Failure Time Data: Kalbfleisch/The Statistical*. John Wiley & Sons, Inc.; 2002. doi:10.1002/9781118032985
10. Kaplan E, Meier P. Nonparametric estimation from incomplete observations. *J Am Stat Assoc*. 1958;53:457-481.
11. Therneau TM, Grambsch PM, Fleming TR. Martingale-based residuals for survival models. *Biometrika*. 1990;77(1):147-160. doi:10.1093/biomet/77.1.147
12. Hosmer DW, Lemeshow S, May S. *Applied Survival Analysis: Regression Modeling of Time-to-Event Data*. 2nd ed.; 2008.
13. Cleveland WS. Robust Locally Weighted Regression and Smoothing Scatterplots. 1979;74(368):829-836.
14. Grambsch PM, Therneau TM. Proportional hazards tests and diagnostics based on weighted residuals. *Biometrika*. 1994;81(3):515-526. doi:10.1093/biomet/81.3.515
15. Schoenfeld D. Partial residuals for the proportional hazards regression model. *Biometrika*. 1982;69(1):239-241. doi:10.1093/biomet/69.1.239
16. Simon N, Friedman J, Hastie T, Tibshirani R. Regularization Paths for Cox’s Proportional Hazards Model via Coordinate Descent. *J Stat Softw*. 2011;39(5):1-13. doi:10.18637/jss.v039.i05
17. Steck H, Krishnapuram B, Dehing-Oberije C. On Ranking in Survival Analysis: Bounds on the Concordance Index. Published online 2008.
18. Blanche P, Dartigues J-F, Jacqmin-Gadda H. Estimating and comparing time-dependent areas under receiver operating characteristic curves for censored event times with competing risks. *Stat Med*. 2013;32(30):5381-5397. doi:10.1002/sim.5958
19. Riley RD, Snell KI, Ensor J, et al. Minimum sample size for developing a multivariable prediction model: PART II - binary and time-to-event outcomes. *Stat Med*. 2019;38(7):1276-1296. doi:10.1002/sim.7992
