# Supplementary material for: Prior fluid and electrolyte imbalance is associated with COVID-19 mortality
Source: Commun Med (Lond). 2021 Nov 25;1:51. doi: 10.1038/s43856-021-00051-x (PMC9053234; doi:10.1038/s43856-021-00051-x)
Supplement: Supplementary file 10 — Description of Additional Supplementary Files [file 43856_2021_51_MOESM10_ESM.pdf]

Description of Additional Supplementary Files

File Name: Supplementary Data 1

Description: Cohort characteristics. Univariable analysis was performed for 122,250 patients that passed quality criteria, and multivariable analysis for 55,757 patients, for whom at least ten variables were available. The number of deceased individuals was 6713 in the univariable cohort and 3306 in the multivariable cohort.

File Name: Supplementary Data 2

Description: Names and results of PCR tests in the Optum® database release of 13 Jul 2020, and whether they were interpreted as positive, negative or indecisive results in the analyses.

File Name: Supplementary Data 3

Description: ICD codes of individual diseases in the comorbidity groups used in the multivariable analysis.

File Name: Supplementary Data 4

Description: Univariable associations of all variables for the entire patient cohort and by age group.

File Name: Supplementary Data 5

Description: Performance measures of the combined, comorbidity, and labs/vitals multivariable models.

File Name: Supplementary Data 6

Description: Analysis of variables that sequestered renal comorbidities, hypertension, and low DBP from the combined multivariable model.

File Name: Supplementary Data 7

Description: Independent variables in the combined, comorbidity, and labs/vitals multivariable models and corresponding univariable analysis results.

File Name: Supplementary Data 8

Description: Univariable analysis of the individual diseases in the fluid, pH and electrolyte imbalance (FPEI) comorbidity group.
